# γ-Aminobutyric acid (GABA) signalling in human pancreatic islets is altered in type 2 diabetes

**DOI:** 10.1007/s00125-012-2548-7

**Published:** 2012-04-27

**Authors:** J. Taneera, Z. Jin, Y. Jin, S. J. Muhammed, E. Zhang, S. Lang, A. Salehi, O. Korsgren, E. Renström, L. Groop, B. Birnir

**Affiliations:** 1 Department of Neuroscience, Uppsala University, Box 593, 75124 Uppsala, Sweden; 2 Lund University Diabetes Center, Department of Clinical Sciences, Diabetes & Endocrinology, University Hospital Malmö, Lund University, Malmö, Sweden; 3 Department of Clinical Sciences, Islet Cell physiology, University Hospital Malmö, Lund University, Malmö, Sweden; 4 Department of Clinical Sciences, Islet Pathophysiology, University Hospital Malmö, Lund University, Malmö, 20502 Sweden; 5 Department of Immunology, Genetics and Pathology, Uppsala University, Uppsala, 75185 Sweden

**Keywords:** γ-Aminobutyric acid, Gene expression, Human islets, Type 2 diabetes

## Abstract

**Aims/hypothesis:**

γ-Aminobutyric acid (GABA) is a signalling molecule in the interstitial space in pancreatic islets. We examined the expression and function of the GABA signalling system components in human pancreatic islets from normoglycaemic and type 2 diabetic individuals.

**Methods:**

Expression of GABA signalling system components was studied by microarray, quantitative PCR analysis, immunohistochemistry and patch-clamp experiments on cells in intact islets. Hormone release was measured from intact islets.

**Results:**

The GABA signalling system was compromised in islets from type 2 diabetic individuals, where the expression of the genes encoding the α1, α2, β2 and β3 GABA_A_ channel subunits was downregulated. GABA originating within the islets evoked tonic currents in the cells. The currents were enhanced by pentobarbital and inhibited by the GABA_A_ receptor antagonist, SR95531. The effects of SR95531 on hormone release revealed that activation of GABA_A_ channels (GABA_A_ receptors) decreased both insulin and glucagon secretion. The GABA_B_ receptor antagonist, CPG55845, increased insulin release in islets (16.7 mmol/l glucose) from normoglycaemic and type 2 diabetic individuals.

**Conclusions/interpretation:**

Interstitial GABA activates GABA_A_ channels and GABA_B_ receptors and effectively modulates hormone release in islets from type 2 diabetic and normoglycaemic individuals.

**Electronic supplementary material:**

The online version of this article (doi:10.1007/s00125-012-2548-7) contains peer-reviewed but unedited supplementary material, which is available to authorised users.

## Introduction

γ-Aminobutyric acid (GABA) is an extracellular signal molecule in the pancreatic islet [1–6]. GAD catalyses the formation of GABA from glutamate in the beta cells where GABA is present in both the cytoplasm and vesicles [1, 6]. Once released, GABA is thought to act in an auto- and para-crine manner on the islet cells to modulate hormone secretion [7, 8]. The effective interstitial physiological concentration of GABA in islets is not known. Keeping the proper levels may be critical for balancing the insulin and glucagon secretion. Interstitial GABA may also have a role in maintaining beta cell mass [9, 10].

GABA activates GABA_A_ receptors and the GABA_B_ receptor in the plasma membrane of alpha, beta and delta cells [8, 11–14]. The GABA_A_ receptors are pentameric ion channels (GABA_A_ channels) and normally contain three types of subunit: 2αs, 2βs and a third type of subunit. To date, 19 different mammalian GABA_A_ subunits (α1-6, β1-3, γ1-3, δ, ε, π, θ and ρ1-3) have been cloned [15, 16]. Evidence for the existence of a multitude of GABA_A_ channel subtypes comes from pharmacological studies. It has been shown that, for example, benzodiazepine-site ligands can differentiate between GABA_A_ channel subtypes based on the type of α and γ subunits in the channel complex [16]. Studies with ligands such as GABA and general anaesthetics have further confirmed and extended the list of GABA_A_ channel subtypes [16]. The heterogeneity of channel subunits not only results in differential response to drugs but also in a range of physiological properties such as variable channel conductance and kinetics [16, 17]. The response to GABA can be further modulated by intracellular proteins and factors that are associated with the channel complex [16, 17]. In contrast, there is only one type of GABA_B_ receptor, which is a G-protein-coupled receptor composed of two homologous subunits, GABABR1 and GABABR2 [18]. The GABAB1 subunit binds GABA, whereas the GABAB2 subunit is responsible for Gi/o-protein-coupled activation [19].

The GABA_A_ channels have been best studied in the brain and can be classified into two main groups, synaptic and extrasynaptic channels, based on their location on neurons and physiological and pharmacological characteristics [16, 17, 20]. Synaptic channels are located in the postsynaptic membrane, respond rapidly and are activated by millimolar concentrations of GABA, whereas extrasynaptic channels are located outside of synapses, are activated with a latency of minutes and are saturated by submicromolar GABA concentrations [17, 20]. In the islets, GABA is released by synaptic-like microvesicles [1], by exocytosis together with insulin from the large-dense core vesicles [8], by kiss-and-run exocytosis [8] or by a non-vesicular process [6, 21]. Only in the vicinity of the vesicular release site on the beta and delta cells can the GABA concentration be expected to be in the tens of micromolar to millimolar range [1, 8] and activate transient (phasic), synaptic-like GABA_A_ currents. Elsewhere, the low interstitial concentrations of GABA may generate long-lasting (tonic) currents by activating extrasynaptic-like GABA_A_ channels in the islet cells similar to that recorded in neurons [20]. If GABA modulates the electrical activity of the islet cells, then the spatial and temporal relative concentration of GABA in the endocrine pancreas has the potential to modulate hormone secretion in the islets and thereby may contribute to the pathogenesis of type 2 diabetes if the GABA concentration fluctuations fall outside of the normal range [4]. Recently, Braun et al showed that many of the 19 GABA_A_ channel subunits are expressed in human pancreatic islets [8]. Here we extend the search to profile the expression of the GABA signalling system in pancreatic islets obtained from individuals with or without type 2 diabetes. We demonstrate that the GABA_A_ channel subunits, α1, α2, β2 and β3, are downregulated in islets from individuals with type 2 diabetes. We also show that the interstitial GABA concentration in intact human islets activates GABA_A_ channel currents, which are increased by pentobarbital. Hormone secretion is altered in islets from individuals with type 2 diabetes but can be enhanced with GABA_A_ and GABA_B_ antagonists.

## Methods

### Human tissue

Islets from cadaver donors were provided by the Nordic Islet Transplantation Programme (www.nordicislets.org). Islets were obtained from 54 non-diabetic donors (25 female, 29 male; mean ± SD: age 59 ± 9 years, BMI 25.9 ± 3.5, HbA_1c_ 5.5 ± 1.1% [37 ± 11 mmol/mol] and days of culture 3.5 ± 1.9) and nine type 2 diabetic donors (four female, five male; mean ± SD age 57 ± 4 years, BMI 28.5 ± 4.5, HbA_1c_ 7.2 ± 1.1%, (55 ± 11 mmol/mol) and days of culture 3.3 ± 1.5). Purity of islets was measured by dithizone staining and varied from 57 ± 19% in the type 2 diabetic islets to 67 ± 17% in the non-diabetic islets (*p* = 0.15). We also tried to obtain an estimate of the contribution of exocrine and endocrine tissue by measuring expression of pancreatic lipase, α2 amylase and chymotrypsin 2, as markers of exocrine tissue, and somatostatin and glucagon, as markers of endocrine tissue (probes for insulin were not on the Affymetrix chip). Using this approach, we found that the contribution of endocrine tissue did not differ between non-diabetic and type 2 donors (72 ± 12% vs 68 ± 10%, *p* = 0.29). We also measured insulin content as a surrogate marker for beta cell mass in pancreatic islets from hyperglycaemic and normoglycaemic donors and observed only a modest insignificant decrease in insulin content in islets from hyperglycaemic vs normoglycaemic donors (4.8 ± 3.2 vs 5.6 ± 3.2; *p* = 0.4). To obtain a measure of the contribution of beta and alpha cells in hyperglycaemic and normoglycaemic individuals, we calculated the ratio between MafA, a beta cell marker, and MafB, an alpha cell marker; this ratio did not differ between hyperglycaemic and normoglycaemic donors (43 ± 13% vs 38 ± 12%, *p* = 0.1). Only hand-picked islet preparations were used for the hormone and electrophysiological experiments. The islets were cultured in CMRL 1066 (ICN Biomedicals, Costa Mesa, CA, USA) supplemented with 10 mmol/l HEPES, 2 mmol/l l-glutamine, 50 μg/ml gentamicin, 0.25 μg/ml Fungizone (Gibco, Gaithersburg, MD, USA), 20 μg/ml ciprofloxacin (Bayer Healthcare, Leverkusen, Germany) and 10 mmol/l nicotinamide at 37°C (5% CO_2,_ vol./vol.) for 1–9 days before being used in experiments. All procedures were approved by the ethics committees at Uppsala and Lund Universities, and informed consent was obtained by appropriate measures from donors or their relatives.

### Total RNA isolation

Total RNA was isolated from islets using the AllPrep DNA/RNA Mini Kit (Qiagen, Hilden, Germany). RNA concentration and quality were measured using a NanoDrop ND-1000 spectrophotometer (NanoDrop Technologies, Wilmington, DE, USA) and Experion RNA gel chips (Bio-Rad, Hercules, CA, USA), respectively. RNA from sorted human pancreatic islet beta cells was kindly provided by Dr Cilio, Lund University.

### Microarray gene expression

The microarrays were performed following the Affymetrix standard protocol as previously described [22] (see electronic supplementary material [ESM] text).

### Quantitative

*PCR (RT-qPCR)* Gene expression profiling of the GABA signalling system components in human islet was performed on total RNA by RT-qPCR as previously described [9] (see ESM text). The primer sequences are shown in ESM Table 1.

### Immunofluorescence staining

Immunofluorescence staining of human islets was performed as previously described [23] (see ESM text).

### Current recordings from cells in intact islets

All patch-clamp recordings were performed at room temperature (20–22°C) on intact islets. Drugs used were purchased from Sigma-Aldrich (Stockholm, Sweden) or Ascent Scientific (Weston-Super-Mare, UK). The positive modulator, pentobarbital, and the GABA_A_ antagonist, SR95531, were dissolved in the extracellular solution. For the whole-cell voltage-clamp recordings, islets were perfused with an extracellular solution containing in mmol/l: 126 NaCl, 5.6 KCl, 2.6 CaCl_2_, 1.2 MgCl_2_ and 10 HEPES, pH 7.4 . The pipette solution contained in mmol/l: 125 CsCl, 30 CsOH, 10 EGTA, 1 MgCl_2_, 5 HEPES, pH 7.2. Recordings were made at the holding potential of −70 or −90 mV. The single-channel recordings were performed in the whole-cell patch-clamp configuration. Patch pipettes were made from borosilicate glass and had a resistance of 5–8 MΩ when filled with the pipette solution. Patch-clamp current recordings were carried out using an Axopatch 200B amplifier, filtered at 2 kHz, digitised online at 10 kHz using an analogue-to-digital converter and analysed with pClamp software (Molecular Devices, Sunnyvale, CA, USA).

### Glucose-stimulated hormone secretion in human islets

Secretion of insulin or glucagon in human islets stimulated by glucose was measured by the standard protocol as previously described [24] (see ESM text).

### Statistical analysis

Data are presented as mean±SEM. Differences in expression levels were analysed by Student’s *t* test or non-parametric Mann–Whitney test. Correlations were analysed using non-parametric Spearman’s tests. In all tests, *p* < 0.05 was considered significant. All statistical tests were performed using SPSS version 18.0 software (SPSS, Chicago, IL, USA) or Sigma Plot version 11 (Systat Software, San Jose, CA, USA).

## Results

### Expression of genes encoding the GABA signalling system components in islets

A comprehensive expression profile of genes encoding proteins participating in the GABA signalling cascade in excitable cells was created using cDNA microarray from islets from 54 non-diabetic donors (ESM Fig. 1a, b). Of the genes encoding the GABA_A_ channel subunits, the β3, γ2, δ, ε, π and ρ2 subunits were most prominently expressed in the islets (ESM Fig. 1a). High expression signals were also detected for *GABARAP* (GABA receptor-associated protein), radixin (*RDX*), the NKCC1 transporter (*SLC12A2*) and *GAD65* (also known as *GAD2*) (ESM Fig. 1b).

**Fig. 1:**
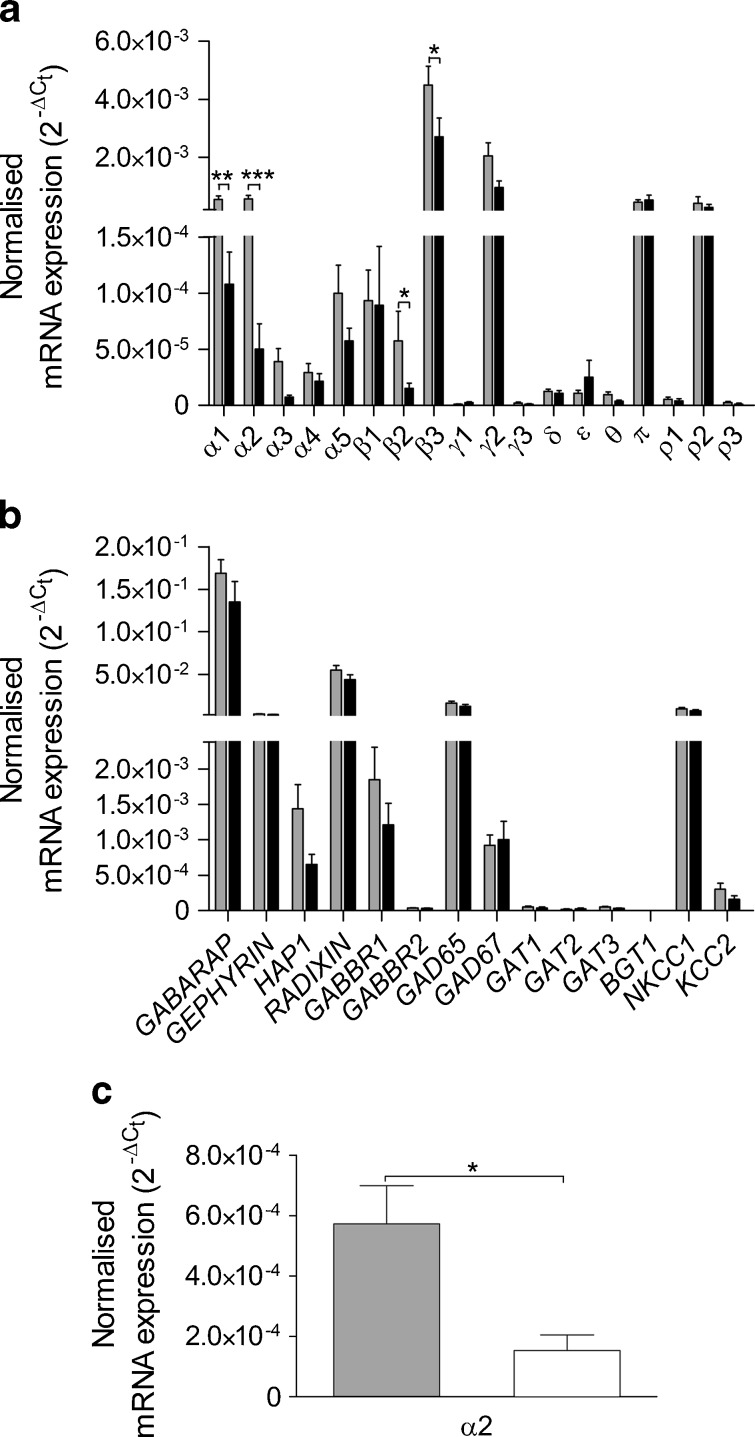
Gene expression of GABA signalling system components in human islets determined by RT-qPCR. **a** In islets from normoglycaemic donors (grey bar, *n* = 14) and donors with type 2 diabetes (black bar, *n* = 9), 18 out of 19 GABA_A_ channel subunits were detected, with the most prominent expression level for α1, α2, β3, γ2, π and ρ2. In islets from individuals with type 2 diabetes, expression of the α1, α2, β2 and β3 GABA_A_ channel subunits was significantly downregulated compared with normoglycaemic donors. **b** Gene expression of GABA signalling system accessory proteins, receptors, enzymes and transporters from normoglycaemic donors (grey bar, *n* = 14) and donors with type 2 diabetes (black bar, *n* = 9). The most prominent expression was detected for *GEPHRYN*, *GABARAP*, *RADIXIN*, *GAD65* and the chloride transporter, *NKCC1* (also known as *SLC12A2*). **c** In islets from hyperglycaemic donors (open bar, *n* = 6), expression of the α2 GABA_A_ channel subunit was downregulated compared with normoglycaemic donors (grey bar, *n* = 14). The relative expression of each target gene was normalised to reference gene *ACTB* using the ${2^{{ - \Delta {{\rm{C}}_{\rm{t}}}}}}$method. All individual experiments were run in duplicate, and the data presented as mean ± SEM. Differences in expression levels were analysed by Student's *t* test: *p < 0.05, **p < 0.01, ***p < 0.001

We further examined and compared gene expression in islets from individuals with and without type 2 diabetes using RT-qPCR analysis (Fig. 1). The highest level of expression was obtained for the genes for α1, α2, β3, γ2, π and ρ2 GABA_A_ channel subunits in islets from normoglycaemic individuals (*n* = 14). In islets from type 2 diabetic donors, the α1, α2, β2 and β3 GABA_A_ subunits were significantly downregulated (Fig. 1a). The other genes in the GABA signalling cascade (Fig. 1b) were all similarly expressed and did not differ significantly between islets from individuals with type 2 diabetes and those from normoglycaemic donors. We further examined whether we could detect downregulation of genes in the islets from hyperglycaemic donors (not diagnosed with type 2 diabetes; *n* = 6). Only the α2 GABA_A_ channel subunit was significantly downregulated in islets from hyperglycaemic individuals compared with normoglycaemic individuals (Fig. 1c, α1, *p* = 0.127; β2, *p* = 0.536; β3, *p* = 0.386).

### Localisation of GABA_A_ α1 and α2 channel subunit proteins in human pancreatic islets

**Fig. 2:**
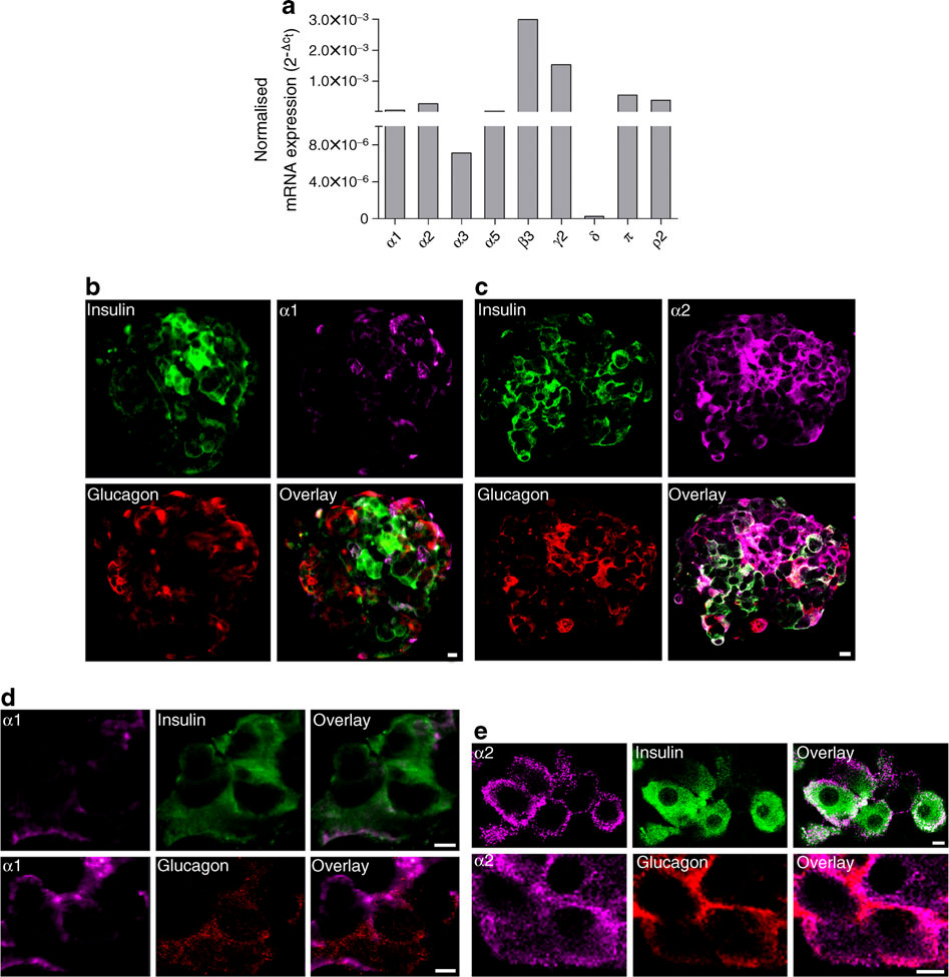
GABA_A_ channel subunit mRNA expression and protein localisation in human pancreatic islet alpha and beta cells. **a** Expression of GABA_A_ channel subunits in sorted human pancreatic islet beta cells from one normoglycaemic donor quantified by RT-qPCR. The β3 and γ2 subunits were prominently expressed, and the expression of the α1, α2, π and ρ2 subunits was somewhat lower. The relative expression of each target gene was normalised to the reference gene *ACTB* using the ${2^{{ - \Delta {{\rm{C}}_{\rm{t}}}}}}$method. **b** Human islets were co-labelled with antibodies staining insulin (green), α1 GABA_A_ channel subunit (purple) and glucagon (red). Scale bar, 5 μm. **c** Human islets were co-labelled with antibodies staining insulin (green), α2 GABA_A_ channel subunit (purple) and glucagon (red). Scale bar, 5 μm. **d** Immunoreactivity of α1 GABA_A_ channel subunit (purple) was detected in both insulin-positive cells (green) and glucagon-positive cells (red) from human islets. Scale bar, 5 μm. **e** Immunoreactivity of α2 GABA_A_ channel subunit (purple) was detected in both insulin-positive cells (green) and glucagon-positive cells (red) from human islets. Scale bar, 5 μm

To determine which specific GABA_A_ channel subunits are present in pancreatic islet beta cells, we analysed expression of the subunits in sorted beta cells from one normoglycaemic donor using RT-qPCR. The α1, α2, α5, β3, γ2, δ, π and ρ2 GABA_A_ channel subunits were present in the cells (Fig. 2a). To identify the cellular and subcellular location of the GABA_A_ channel subunits, we immunostained for the α1 or α2 subunits together with insulin and glucagon in pancreatic islets (Fig. 2b–e). The results show that, in human pancreatic islets, the α1 and the α2 GABA_A_ channel subunit proteins are present in the plasma membrane and cytoplasm of alpha and beta cells. The α2 subunit appears particularly prominent in the cells, whereas α1 expression is more limited.

### Interstitial GABA activates GABA_A_ channel currents in intact islets

**Fig. 3:**
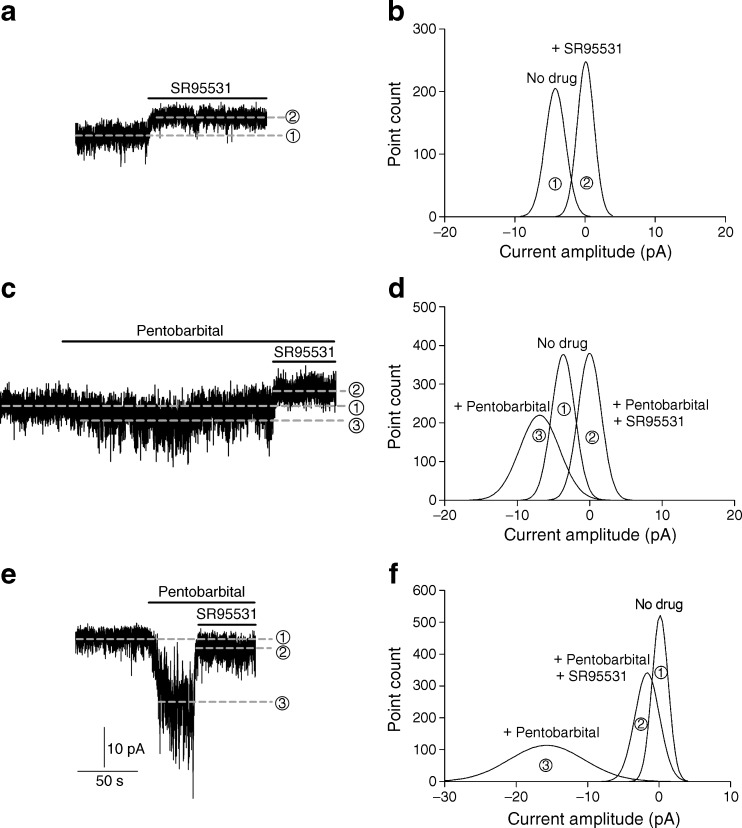
Activation, by interstitial GABA, of GABA_A_ channel currents that are enhanced by pentobarbital in human pancreatic islets. **a** A representative current trace showing GABA-activated tonic current, recorded from a cell in an intact human islet from a normoglycaemic donor and activated by interstitial GABA, was inhibited by application of SR95531 (100 μmol/l), a GABA_A_ channel antagonist, causing a clear outward shift in the holding current (line 1 to line 2). SR95531 similarly inhibited the baseline current in islet cells in another four experiments. **b** Gaussian fits to all-point histograms derived from 30 s current recordings in (**a**) before (1) and during (2) application of SR95531. The difference between the peaks of the Gaussian fits denotes the mean tonic current (−4.4 pA). **c** A representative current trace showing that pentobarbital (100 μmol/l) increased the GABA-activated tonic current (inward shift of the holding current line 1 to line 3), recorded from a cell in an intact human islet from a normoglycaemic donor and activated by the interstitial GABA. The current was then inhibited by 100 μmol/l SR95531 (outward shift of the holding current line 3 to line 2). Pentobarbital similarly enhanced the GABA-generated current in islet cells in another five experiments. **d** Gaussian fits to all-point histograms derived from 30 s current recordings in (**c**) before (1) and during application of pentobarbital (3) or pentobarbital together with SR95531 (2). The peak of the Gaussian fits denotes the mean holding currents (1, −3.6 pA; 2, 0 pA; 3, −7.1 pA). **e** A representative current trace showing that pentobarbital (100 μmol/l) increased the GABA-activated tonic current (inward shift of the holding current line 1 to line 3), recorded from a cell in an intact human islet from a type 2 diabetes donor and activated by the interstitial GABA. The current was then partially inhibited by 100 μmol/l SR95531 (outward shift of the holding current line 3 to line 2). **f.** Gaussian fits to all-point histograms derived from 30 s current recordings in (**e**) before (1) and during application of pentobarbital (3) or pentobarbital together with SR95531 (2). The peak of the Gaussian fits denotes the mean holding currents (1, 0 pA; 2, −2.1 pA; 3, −16.1 pA). Currents were recorded in 20 mmol/l glucose from cells in intact islets at the holding potential of −90 mV in the whole-cell patch-clamp configuration. As no GABA was added experimentally, the GABA must have originated within the islet

**Fig. 4:**
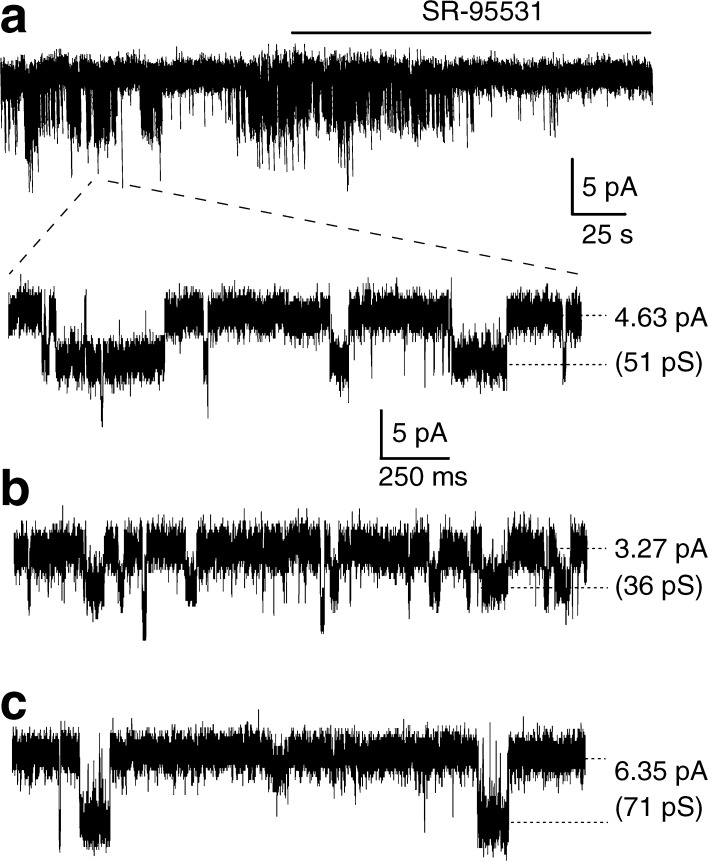
Interstitial GABA activates single-channel currents in intact islets. Single-channel currents that were later inhibited by 100 μmol/l SR95531 were recorded in three different cells (**a**, **b**, **c**) from normoglycaemic donors. **a** A representative current trace (top trace, slow time scale, s) showing GABA-activated single-channel currents and inhibition by 100 μmol/l SR95531. The solid line shows the time when the extracellular solution containing SR95531 was perfused through the recording chamber. The broken lines indicate from where in the recording the current trace on the faster time scale (ms) was obtained. The glucose concentration was 20 mmol/l and the holding potential was −90 mV (**a**), −70 mV (**b**) and −70 mV (**c**). The most prominent single-channel conductance in the cells in **a**, **b** and **c** was 51 pS, 36 pS and 71 pS, respectively. For all three cells, the currents were recorded in the whole-cell patch-clamp configuration from cells in intact islets and were activated by interstitial GABA and inhibited by application of SR95531 (100 μmol/l)

The exact interstitial GABA concentration in the islets is not known, but can be assumed to be in the submicromolar range or similar to that in the extracellular fluid in the brain [25]. The highest GABA concentration is expected to be around the beta cell release sites. Using the patch-clamp technique and recording from cells in intact islets, we examined currents from GABA_A_ channels activated by the interstitial GABA originating within the islets, as no GABA was added experimentally. Figure 3a shows whole-cell currents, which were inhibited by the GABA_A_ channel competitive antagonist, SR95531 (100 μmol/l). The upward shift in the baseline current when SR95531 was applied shows the level of the GABA-activated GABA_A_ current. The levels of lines 1 and 2 in Fig. 3a correspond to the peak values (Fig. 3b) of Gaussian fits to histograms of 30 s current records from before and after SR95531 application. The difference between the peak values was 4.4 pA and is the level of the GABA-generated current in the cell. This type of current is termed ‘tonic’, as it is long lasting and can significantly affect cell excitability [20]. In two cells, synaptic-like transient currents were recorded (data not shown) similar to that reported by Braun et al [1, 8]. We examined whether larger tonic currents were generated if we applied no glucose to the cells, but applied 10 mmol/l glutamine. Under these conditions, no tonic currents were recorded (*n* = 5), but when we applied 20 mmol/l glucose to islets from the same donor, tonic currents were evoked (*n* = 3). We then examined whether a positive modulator of GABA_A_ channels, pentobarbital, enhanced the GABA-generated tonic currents (Fig. 3c, d). The level of line 3 (Fig. 3c) corresponds to the peak value (Fig. 3d) of the Gaussian fit to a histogram of 30 s current record after 100 μmol/l pentobarbital application to the islet. The level of the GABA-generated tonic current in the cell was 3.6 pA and was enhanced to 7.1 pA by 100 μmol/l pentobarbital (Fig. 3d). Interestingly, GABA-generated tonic currents were minimal or not detected in islets from type 2 diabetic donors until we applied pentobarbital (*n* = 5, Fig. 3e, f). The results are consistent with pentobarbital enhancing GABA_A_ currents by increasing both the open probability and the channel conductance of GABA_A_ channels, resulting in higher apparent affinity of the channels for GABA. The enhanced current was inhibited by SR95531 (Fig. 3e, f). We recorded single-channel currents from three different cells (Fig. 4a, b, c) in islets from normoglycaemic donors (Fig. 4a, slow time scale (s); Fig. 4a expanded, Fig. 4b and Fig. 4c, fast time scale (ms); the glucose concentration was 20 mmol/l and the holding potential: Fig. 4a, −90 mV; Fig. 4b, −70 mV; Fig. 4c, −70 mV). In all three cells, when the islets were perfused with 100 μmol/l SR95531, the single-channel currents were inhibited. Figure 4a (top current trace, time scale s) shows SR95531 inhibition of the channels in one of the cells. The most prominent single-channel current amplitude recorded in each cell is indicated in Fig. 4 by the dotted lines and gave channel conductance of 51 pS, 36 pS and 71 pS for the cells in Fig. 4a, b and c, respectively.

### Effects of GABA_A_ channel and GABA_B_ receptor antagonists on insulin and glucagon secretion in islets from individuals with or without type 2 diabetes

**Fig. 5:**
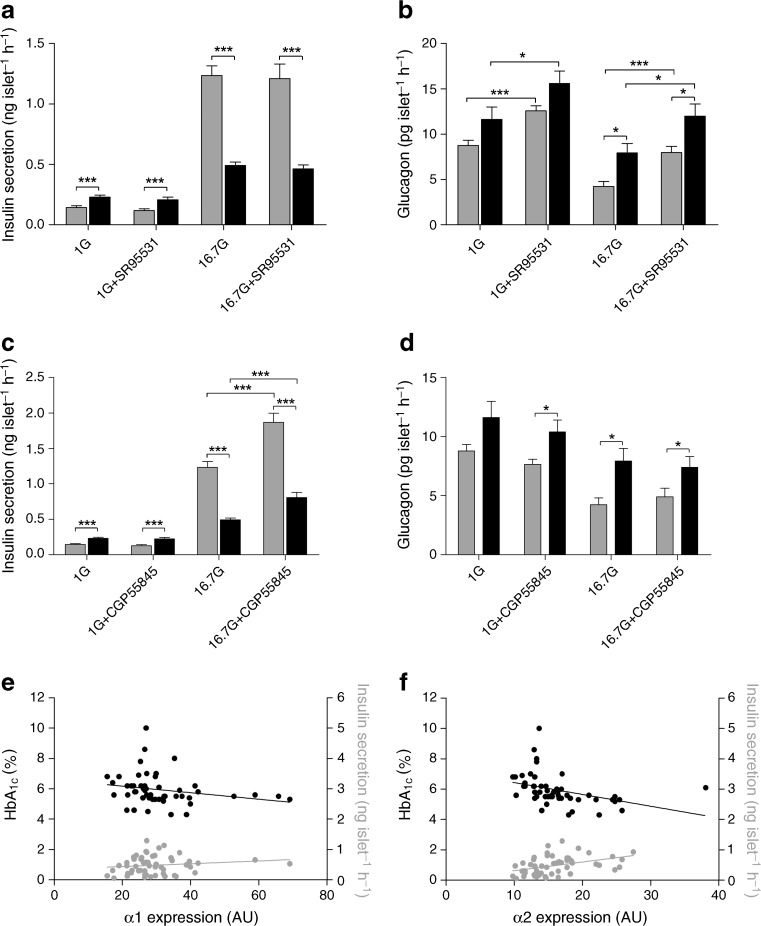
GABA_A_ channel and GABA_B_ receptor antagonists increase hormone release in pancreatic islets from normoglycaemic and type 2 diabetic individuals. **a** Insulin release was increased in 1 mmol/l glucose (1G) but decreased in 16.7 mmol/l glucose (16.7G) in islets from type 2 diabetic individuals (black bar) compared with those from normoglycaemic individuals (grey bar). SR95531, a GABA_A_ channel antagonist, at a concentration of 10 μmol/l did not modulate insulin release. **b** Glucagon release was increased in 16.7 but not 1 mmol/l glucose in islets from type 2 diabetic individuals compared with those from normoglycaemic donors. SR95531 (10 μmol/l) increased glucagon release at both glucose concentrations in islets from type 2 diabetic and normoglycaemic individuals. **c** Insulin release was increased by the GABA_B_ antagonist, CGP55845 (10 μmol/l) in 16.7 but not 1 mmol/l glucose in islets from type 2 diabetic and normoglycaemic individuals. **d** Glucagon release was not affected by CGP55845 (10 μmol/l). Grey bars: *n* = 19–31 from four to six normoglycaemic individuals. Black bars: *n* = 19 from three type 2 diabetic donors. Data are presented as mean ± SEM; *p < 0.05, ***p < 0.001. **e** Correlation of the α1 GABA_A_ subunit gene expression with insulin secretion measured at 16.7 mmol/l glucose and HbA_1c_ level. A negative correlation with HbA_1c_ (black circles; *n* = 51; *R* = 0.3724; *p* = 0.0071) and no correlation with insulin (grey circles; *n* = 53; *R* = 0.1655; *p* = 0.2363) were observed. Correlation analysis was performed using non-parametric Spearman’s test. **f** Correlation of the α2 GABA_A_ subunit gene expression with insulin secretion measured at 16.7 mmol/l glucose and HbA_1c_ level. A negative correlation with HbA_1c_ (black circles; *n* = 51; *R* = 0.6453; *p* < 0.0001) and positive correlation with insulin (grey circles; *n* = 53; *R* = 0.4790; *p* = 0.0003) were observed. Correlation analysis was performed using non-parametric Spearman’s test. NB To convert values for HbA_1c_ in % into mmol/mol, subtract 2.15 and multiply by 10.929

In islets from both normoglycaemic and type 2 diabetic donors, 16.7 mmol/l glucose stimulated insulin secretion and reduced glucagon secretion relative to the secretion observed in response to 1 mmol/l glucose (ESM Fig. 2). To study the effects of GABA on insulin and glucagon release, we examined the effects of the GABA_A_ and GABA_B_ antagonists, SR95531 (10 μmol/l, Fig. 5a, b) and CGP55845 (10 μmol/l, Fig. 5c, d), on hormone secretion in islets from normoglycaemic and type 2 diabetic individuals incubated with basal (1 mmol/l) or high-concentration (16.7 mmol/l) glucose.

Figure 5a and c show that insulin secretion was increased at 1.0 mmol/l but significantly decreased at 16.7 mmol/l glucose in islets from individuals with type 2 diabetes compared with islets from normoglycaemic donors. At 1 mmol/l glucose, insulin secretion was increased by a factor of 1.6 in the islets from type 2 diabetic donors and was unaffected by either SR95531 (10 μmol/l, Fig. 5a) or CGP55845 (Fig. 5c). At 16.7 mmol/l glucose, insulin secretion in islets from the type 2 diabetic individuals was decreased by 60% (Fig. 5a), but could be increased about 1.5-fold with the GABA_B_ receptor antagonist CPG55845 (Fig. 5c) in islets from both type 2 diabetic and normoglycaemic donors. As 10 μmol/l SR95531 may not completely inhibit very high-affinity GABA_A_ channels, we examined in islets from two normoglycaemic individuals whether 100 μmol/l SR95531 had any effect on insulin secretion. In low (1 mmol/l) glucose, 100 μmol/l SR95531 significantly increased insulin secretion (1G + SR100 μmol/l: 0.20 ± 0.03 ng islet^−1^ h^−1^, *n* = 12 from two individuals; 1G: 0.14 ± 0.01 ng islet^−1^ h^−1^, *n* = 12 from four individuals, *p* < 0.05) to the level observed for type 2 diabetic individuals (0.23 ± 0.02 ng islet^−1^ h^−1^, *n* = 12 from three individuals), but failed to modulate insulin secretion in 16.7 mmol/l glucose (*n* = 12 from two individuals, data not shown).

Figure 5b and d show that glucagon secretion was only significantly increased in islets from type 2 diabetic individuals compared with normoglycaemic donors when the islets were exposed to 16.7 mmol/l glucose. Glucagon secretion could be enhanced with 10 μmol/l SR95331 (Fig. 5b) in islets exposed to either 1 or 16.7 mmol/l glucose, whereas CGP555845 (Fig. 5d) was without effect. SR95531 increased glucagon secretion at 1.0 and 16.7 mmol/l glucose by a factor of 1.3 and 1.5, respectively, in islets from type 2 diabetic donors, and a factor of 1.4 and 1.9, respectively, in islets from normoglycaemic donors. The somewhat reduced effect of 10 μmol/l SR95531 on islets from type 2 diabetic individuals may be due to the fact that these islets exhibited increased basal glucagon secretion compared with the normoglycaemic donors. The GABA_B_ receptor antagonist, CGP55845, did not modulate glucagon secretion at either low or high glucose concentrations (Fig. 5d). Together these results demonstrate regulation of hormone release by the GABA_A_ channels and GABA_B_ receptors.

We further examined whether the mRNA expression of GABA_A_ channel subunits α1 and α2 in islets correlated with the HbA_1c_ or insulin secretion of donors. Interestingly, expression of the GABA_A_ channel subunits, α1 and α*2*, in islets correlated negatively with the HbA_1c_ level of donors (Fig. 5e, f), and α2, but not α1, correlated positively with insulin secretion stimulated with 16.7 mmol/l glucose (Fig. 5e, f).

## Discussion

Our results show an active role for the GABA signalling system in human pancreatic hormone release. Interstitial GABA released from pancreatic beta cells evokes GABA-generated currents in intact human islets by activating GABA_A_ channels expressed in alpha and beta cells. The currents can be enhanced by the GABA_A_ channel modulator, pentobarbital, and blocked by the specific GABA_A_ antagonist, SR95531. The α1, α2, β2 and β3 GABA_A_ channel subunit genes are downregulated in islets from type 2 diabetic individuals, whereas other components of the GABA signalling system are not affected. In islets from type 2 diabetic and normoglycaemic individuals, the GABA_B_ receptor antagonist, CPPG55845, increased insulin release at 16.7 mmol/l glucose, whereas the GABA_A_ channel antagonist, SR95531, increased glucagon release in both 1 and 16.7 mmol/l glucose.

Eighteen of 19 possible GABA_A_ channel subunit genes were expressed in the islets, with the highest expression levels detected for the α1, α2, β3, γ2, π and ρ2 subunits. The results are in good agreement with the results of Braun et al [8]. Immunostaining of islets confirmed the presence of α1 and α2 subunits in both the alpha and beta cells. The GABA_A_ channels contain two αs, two βs and a third type of subunit in the channel complex. In islets, γ2, π or ρ2 could each be the third type of subunit in the pentameric channel, but would confer vastly different pharmacological properties on the channel [16]. Intriguingly, the high expression of π and ρ2 subunit genes seen in human islets is very different from the low and restricted distribution in the brain [16, 26].

It is likely that a number of different subtypes of GABA_A_ channels are expressed in the islets and that they differ in their sensitivity to GABA and other drugs [16]. Whether the different single-channel conductances we recorded in this study are related to different GABA_A_ channel subtypes or activation of a specific GABA_A_ channel subtype by different concentrations of GABA remains to be determined [20, 27].

Glucose modulated GABA_A_ channel gene expression is in agreement with previous reports [14], but, in contrast with mice, stimulation with high glucose concentration reduced the α2 GABA_A_ channel subunit mRNA level in human islets. The glucose effect on expression was even stronger in islets from type 2 diabetic individuals, in whom not only α2 but also α1, β2 and β3 GABA_A_ channel subunit expression was decreased compared with islets from normoglycaemic donors. In islets from type 2 diabetic and normoglycaemic individuals, we further compared gene expression of proteins that might modify the function of the GABA signalling system in the islets [17, 28]. These proteins included: (1) the GABA_B_ receptor subunits; (2) the intracellular proteins, gephyrin and radixin, involved in plasma membrane clustering of GABA_A_ channels; (3) GABARAP and HAP1, which take part in transport or recycling of the channels; (4) GAD65 and GAD67, which make GABA from glutamate; (5) GABA transporters (GAT1-3, BGT1), which transport GABA over the cell plasma membrane; and (6) transporters such as NKCC1 and KCC2, which determine the intracellular chloride concentration and thereby determine whether opening of the GABA_A_ channels will cause depolarisation or hyperpolarisation of the cells. None of these proteins was differentially expressed in islets from non-diabetic or type 2 diabetic individuals.

It is well established that GABA can activate GABA_A_ channels in pancreatic islets, but so far it has not been shown that the interstitial GABA concentration that exists within the islets is sufficient to activate the native channels [8, 12]. In recent years, evidence has emerged that extrasynaptic-like GABA_A_ channels can be supersensitive to GABA, being activated by GABA concentrations in the picomolar to nanomolar range [20, 27]. Activation of the channels induces long-lasting (tonic) currents in neurons that modulate the cellular excitability [17, 29, 30]. In this study of intact human islets, interstitial GABA generated tonic currents in the cells. As no GABA was added, the GABA must have originated from cells within the islets. Furthermore, since the GABA-activated current was enhanced by pentobarbital, the interstitial GABA concentration must have been sub-saturating [31]. Whether the reduced level of GABA_A_ channel subunits, the decreased extracellular GABA, or both resulted in the reduced tonic current in the islets from the type 2 diabetic donors remains to be determined. Expression levels for the GABA transporters were low in the islets. In the brain, these transporters are essential for removing GABA from the synapse to avoid inactivation of the GABA_A_ channels [32], but may not be needed in the islets, as small molecules diffuse down their concentration gradient and enter the blood in the highly vascularised islets.

At low (1 mmol/l) glucose, basal GABA inhibits insulin secretion by activating GABA_A_ channels expressed in the beta cells. The finding that a saturating concentration (100 μmol/l) of the antagonist, SR95531, was required to increase the insulin release is consistent with either very high-affinity GABA_A_ channels and/or higher local GABA concentrations in the vicinity of beta cells. How the inhibition comes about may be related to decreased open probability of Ca^2+^ channels by subthreshold depolarisations of the membrane potential for channel activation [33] or a general effect of voltage-dependent inactivation of channels involved in action potential firing [34]. At 16.7 mmol/l glucose, the GABA_B_ antagonist CPG55845 increased insulin release by a factor of 1.5 in islets from both normoglycaemic and type 2 diabetic individuals. These results indicate that the mechanism by which GABA_B_ receptors modulate insulin secretion was intact in islets from type 2 diabetic individuals. They are also in agreement with the unchanged expression levels of GABA_B_ receptor subunits in islets from type 2 diabetic donors.

### Conclusions

The GABA signalling system is compromised in pancreatic islets from type 2 diabetic individuals. The α2 GABA_A_ channel subunit is already downregulated in islets from hyperglycaemic individuals, and, as the disease progresses to type 2 diabetes, expression of the α1, β2 and β3 GABA_A_ channel subunit genes also decreases. The GABA released within the islets normally activates GABA_A_ channels and the GABA_B_ receptor, which modulate insulin and glucagon release. The GABA signalling system is an integral part of a system in human islets maintaining glucose homeostasis. Characterising the functional and pharmacological properties of the GABA_A_ channel subtypes expressed in alpha and beta cells may reveal new specific targets for drugs aimed at modulating hormone release.

## Electronic supplementary material

Below is the link to the electronic supplementary material.

ESM Fig. 1 (PDF 161 kb)

ESM Fig. 2 (PDF 114 kb)

ESM Table 1 (PDF 47 kb)

## Abbreviations

GABA: γ-Aminobutyric acid
qPCR: Quantitative PCR
