# Supplementary material for: γ-Aminobutyric acid (GABA) signalling in human pancreatic islets is altered in type 2 diabetes
Source: Diabetologia. 2012 Apr 27;55(7):1985–94. doi: 10.1007/s00125-012-2548-7 (PMC3369140; doi:10.1007/s00125-012-2548-7)
Supplement: Supplementary file 1 — (PDF 161 kb) [file 125_2012_2548_MOESM1_ESM.pdf]

ESM Figure 1. Microarray gene expression of GABA signalling system components in human pancreatic islets.

(a) Microarray gene expression profile showing mean expression of 19 $GABA_A$ channel subunit genes in pancreatic islets from non-diabetic individuals (grey bar, n = 54; 31 males, 23 females). Abundant expression of the β3, γ2 and π $GABA_A$ channel subunits was observed. Microarrays data were summarized and normalized with Robust Multi-array Analysis (RMA) and presented as mean±SEM. AU, arbitrary units.

(b) Microarray gene expression profile showing mean expression of 14 GABA-signalling system associated proteins, receptors, enzymes and transporter genes in pancreatic islets from non-diabetic individuals (grey bar, n = 54; 31 males, 23 females). Abundant expression of the *GABARAP*, *RADIXIN*, *GAD65* and the *NKCC1* genes was observed.

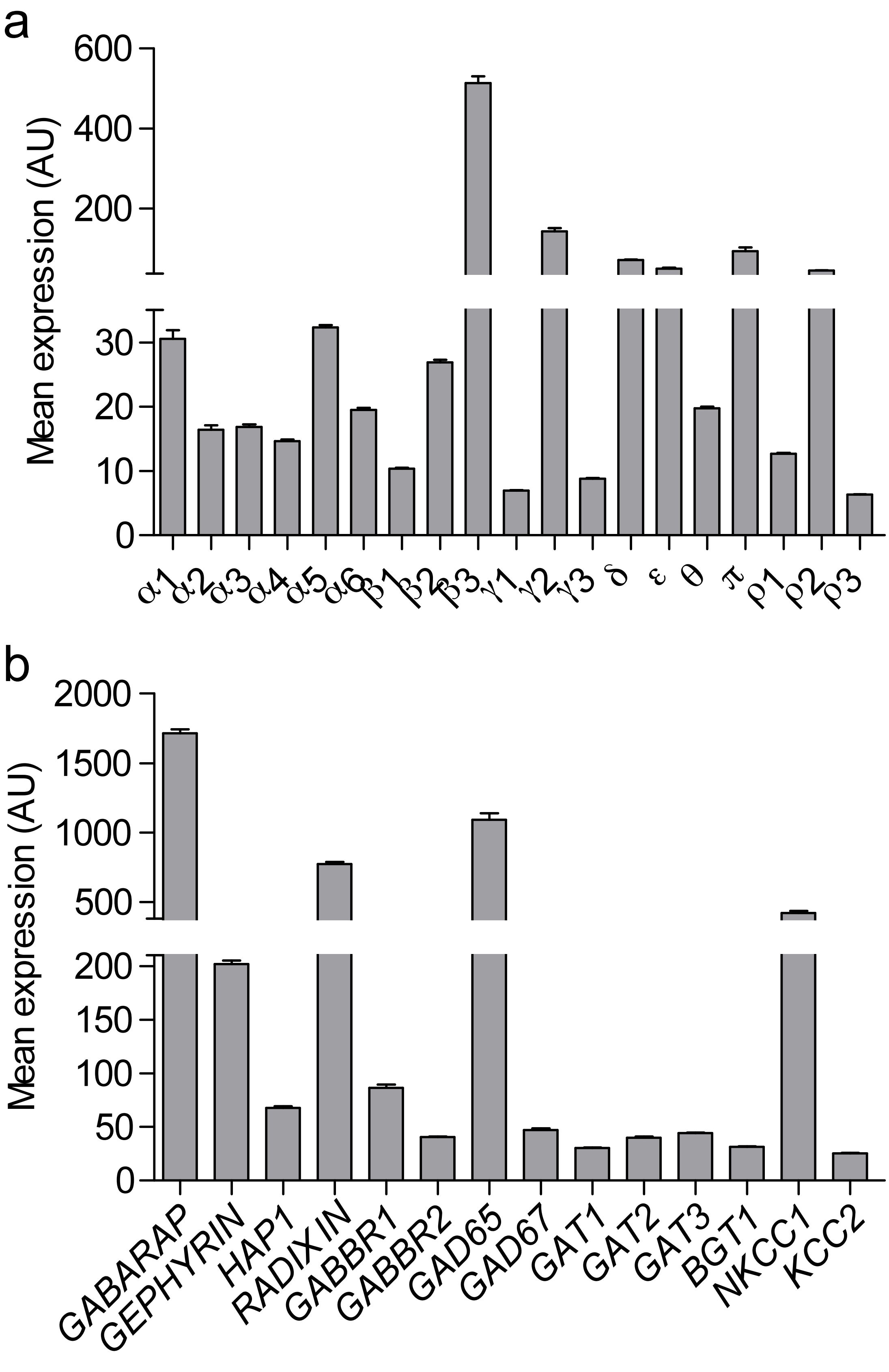
a
Mean expression (AU)
600
400
200
30
20
10
0
α1
α2
α3
α4
α5
α6
β1
β2
β3
γ1
γ2
γ3
δ
ε
θ
π
ρ1
ρ2
ρ3
b
Mean expression (AU)
2000
1500
1000
500
200
150
100
50
0
GABARAP
GEPHYRIN
HAP1
RADIXIN
GABBR1
GABBR2
GAD65
GAD67
GAT1
GAT2
GAT3
BGT1
NKCC1
KCC2
