# Supplementary material for: γ-Aminobutyric acid (GABA) signalling in human pancreatic islets is altered in type 2 diabetes
Source: Diabetologia. 2012 Apr 27;55(7):1985–94. doi: 10.1007/s00125-012-2548-7 (PMC3369140; doi:10.1007/s00125-012-2548-7)
Supplement: Supplementary file 2 — (PDF 114 kb) [file 125_2012_2548_MOESM2_ESM.pdf]

ESM Figure 2. Glucose stimulated insulin secretion and and inhibited glucagon secretion in isolated pancreatic islets from normoglycemic and type 2 diabetic individuals.

(a) Insulin release was significantly increased in 16.7 mmol/l glucose as compared to 1 mmol/l glucose, in islets from both normoglycemic (grey bar) and type 2 diabetic (black bar) individuals.

(b) Glucagon release was significantly decreased in 16.7 mmol/l glucose as compared to in 1 mmol/l glucose, in islets from both normoglycemic and type 2 diabetic individuals.

Data were presented as mean with SEM. *, $P < 0.05$, ***, $P < 0.001$.

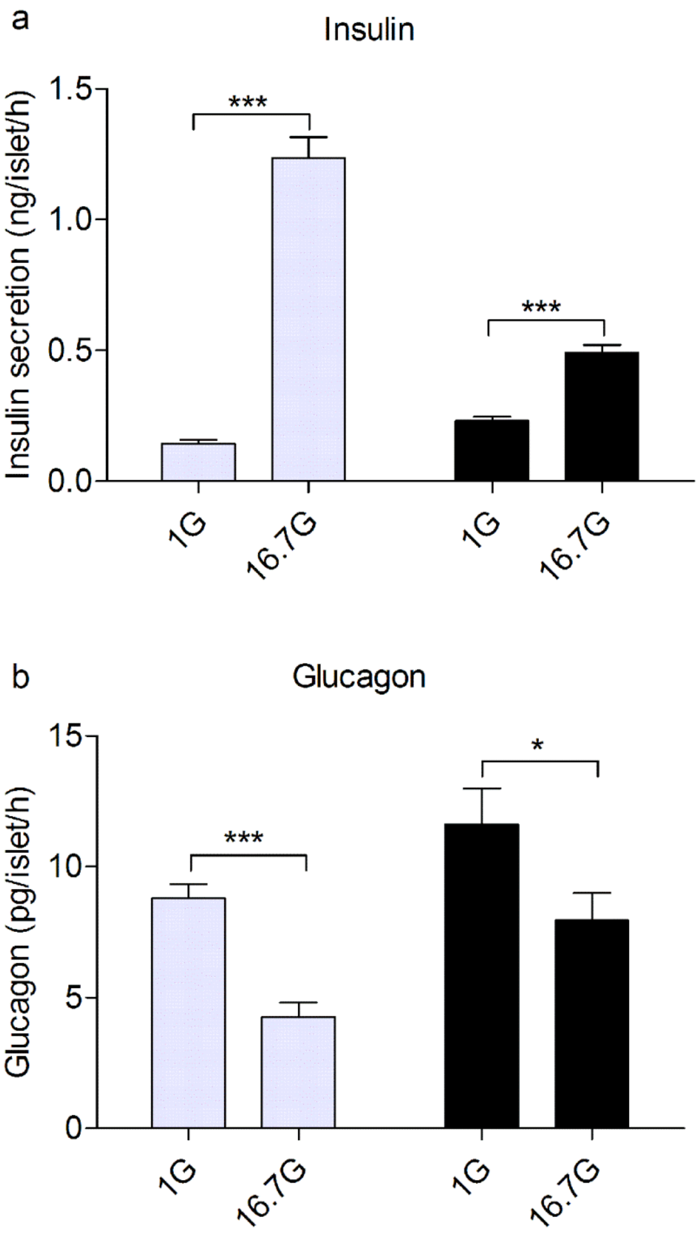
a
Insulin
Insulin secretion (ng/islet/h)
1.5
1.0
0.5
0.0
***
***
1G
16.7G
1G
16.7G
b
Glucagon
Glucagon (pg/islet/h)
15
10
5
0
***
*
1G
16.7G
1G
16.7G
