# Supplementary material for: γ-Aminobutyric acid (GABA) signalling in human pancreatic islets is altered in type 2 diabetes
Source: Diabetologia. 2012 Apr 27;55(7):1985–94. doi: 10.1007/s00125-012-2548-7 (PMC3369140; doi:10.1007/s00125-012-2548-7)
Supplement: Supplementary file 3 — (PDF 47 kb) [file 125_2012_2548_MOESM3_ESM.pdf]

ESM Table 1. Primer sequences used for qRT-PCR.

Gene	Primer	Product size (bp)	Reference Number
α1 (*GABRA1*)	F: GGATTGGGAGAGCGTGTAACC	66	NM_000806
	R: TGAAACGGGTCCGAAACTG		
α2 (*GABRA2*)	F: GTTCAAGCTGAATGCCCAAT	160	NM_000807
	R: ACCTAGAGCCATCAGGAGCA		
α3 (*GABRA3*)	F: CAACTTGTTTCAGTTCATTCATCCTT	102	NM_000808
	R: CTTGTTTGTGTGATTATCATCTTCTTAGG		
α4 (*GABRA4*)	F: TTGGGGGTCCTGTTACAGAAG	105	NM_000809
	R: TCTGCCTGAAGAACACATCCA		
α5 (*GABRA5*)	F: CTTCTCGGCGCTGATAGAGT	105	NM_000810
	R: CGCTTTTTCTTGATCTTGGC		
α6 (*GABRA6*)	F: ACCCACAGTGACAATATCAAAAGC	67	NM_000811
	R: GGAGTCAGGATGCAAAACAATCT		
β1(GABRB1)	F: GTACAAAATCGAGAGAGTCTGGG	144	NM_000812
	R: GCG AAT GTC ATA TCC TTT GAG CA		
β2(*GABRB2*)	F: GCAGAGTGTCAATGACCCTAGT	137	NM_021911
	R: TGGCAATGTCAATGTTCATCCC		
β3(*GABRB3*)	F: CAAGCTGTTGAAAGGCTACGA	108	NM_000814
	R: ACTTCGGAAACCATGTCGATG		
γ1(*GABRG1*)	F: CCTTTTCTTCTGCGGAGTCAA	91	NM_173536
	R: CATCTGCCTTATCAACACAGTTTCC		
γ2(*GABRG2*)	F: CACAGAAAATGACGGTGTGG	136	NM_000816
	R: TCACCCTCAGGAACTTTTGG		
γ3(*GABRG3*)	F: AACCAACCACCACGAAGAAGA	113	NM_033223
	R: CCTCATGTCCAGGAGGGAAT		
δ (*GABRD*)	F: ACCACGGAGCTGATGAACTT	109	NM_000815
	R: AGGGCATGTAGGATTGGATG		
ε (*GABRE*)	F: TGGATTCTCACTCTTGCCCTCTA	107	NM_004961
	R: GGAGTTCTTCTCATTGATTTCAAGCT		
θ (*GABRQ*)	F: CCAGGGTGACAATTGGCTTAA	63	NM_018558
	R: CCCGCAGATGTGAGTCGAT		
π (*GABRP*)	F: GGCCTTGCTAGAATATGCAGTTG	76	NM_014211
	R: CTTTGTTGTCCCCCTATCTTTGG		
ρ1(*GABRR1*)	Hs00266687_m1 from AppliedBiosystem	94	NM_002042
ρ2 (*GABRR2*)	F: CCTAGAAGAGGGCATAGACATCG	99	NM_002043
	R: TCCAGTAGCTGCTGCATTGTTTG		
ρ3 (*GABRR3*)	F: TGATGCTTTCATGGGTTTCA	111	NM_001105580
	R: CGCTCACAGCAGTGATGATT		
GABArap *(GABARAP)*	F: AGGCTCCCAAAGCTCGGATA	100	NM_007278
	R: AATTCGCTTCCGGATCAAGA		
Gephyrin (*GPHN*)	F: TGATCCTTACTAACCACGACCA	97	NM_020806
	R: TTTATCCCACTGCGGTCTTC		
Hap1 (*HAP1*)	F: GAGGCCTCTCAACTCGACAC	66	NM_003949
	R: CTGCTCCACACACTCCAGAA		
Radixin (*RDX*)	F: TGGAACGTCTAAAACAAATTGAAG	106	NM_002906

	R: TTTGCTCGTTTTCGTTCTTG		
GAT1 (*SLC6A1*)	F: GGGAGCTACAACTCTTTCCACAAC	66	NM_003042.2
	R: CGAATTGATGCAGCAGACGAT		
GAT2 (*SLC6A13*)	F: TCTGTGTGGCTTGGGTTTACG	66	NM_016615.2
	R: TGTACCCAATCATGTCTTCGATGT		
GAT3 (*SLC6A11*)	F: ATGCCACCTCCCCTGTCAT	67	NM_014229.1
	R: TGCTCGATCCCGTCAGAGAT		
BGT1 (*SLC6A12*)	F: ACGACCTGCAACAACTTTTGG	62	NM_003044.2
	R: GCTCCTGAGTGGTTCAGAAAGTC		
GABBR1	F: TGGCATGGACGCTTATCGA	78	NM_001470.2
	R: GATCATCCTTGGTGCTGTCATAGT		
GABBR2	F: CTGGTATTCGTGCCGAAGCT	76	NM_005458.6
	R: TGAACTGGAATCGCCTGTTCT		
GAD65 (*GAD2*)	F: CTACGCGTTTCTCCATGCAA	63	NM_000818
	R: GCCAAAGTGGGCCTTTCTC		
GAD67 (*GAD1*)	F: CACCAGTTGCTGGAAGGCAT	124	NM_000817
	R: GAGGATGACCTGTGCGAACC		
NKCC1(*SLC12A2*)	F: ATCAATTTTTCAGTATTCCATGCATC	51	NM_001046
	R: ACGCCATCCTGGAGATTTTG		
KCC2 (*SLC12A5*)	F: CAAGGGTCCAACTTTTCCTG	152	NM_001134771
	R: GCCTCTCGGTTTCTTCCTCT		
β-actin (*ACTB*)	F: CCTGGCACCCAGCACAAT	144	NM_001101.2
	R: GGGCCGGACTCGTCATACT		
